# New strategies for lung cancer diagnosis and treatment: applications and advances in nanotechnology

**DOI:** 10.1186/s40364-024-00686-7

**Published:** 2024-11-13

**Authors:** Jiaqi Feng, Pengpeng Zhang, Dingli Wang, Yuting Li, Jiaxiong Tan

**Affiliations:** 1 WeiFang People’s Hospital, Shandong Second Medical University, Weifang, China; 2 Department of Lung Cancer, Tianjin Lung Cancer Center, Key Laboratory of Cancer Prevention and Therapy, National Clinical Research Center for Cancer, Tianjin’s Clinical Research Center for Cancer, Tianjin Medical University Cancer Institute and Hospital, Tianjin, China; 3 Key Laboratory of Cancer Prevention and Therapy, National Clinical Research Center for Cancer, Tianjin’s Clinical Research Center for Cancer, Tianjin Medical University Cancer Institute and Hospital, Tianjin, China

**Keywords:** Lung cancer, Nanotechnology, Diagnosis, Treatment, Nanoparticles, Clinical innovation

## Abstract

Lung cancer leads in causing cancer-related mortality worldwide, continually posing a significant threat to human health. Current imaging diagnostic techniques, while offering non-invasive detection, suffer from issues such as insufficient sensitivity and the risks associated with radiation exposure. Pathological diagnosis, the gold standard for confirmation, also faces challenges like invasiveness and high costs. In treatment, surgery, radiotherapy, and chemotherapy are the main modalities, each encountering challenges related to precision, environmental adaptability, and side effects. Nanotechnology’s advancement provides new solutions for the diagnosis and treatment of lung cancer, promising to enhance diagnostic accuracy and reduce side effects during treatment. This article introduces the main types of nanomaterials used in the field of lung cancer, offering a comprehensive overview of current research on the application of nanotechnology in early screening, diagnosis, treatment, and monitoring of lung cancer, and summarizing ongoing clinical research findings.

## Introduction

Lung cancer has consistently posed a significant threat to human health, with approximately 2.2 million new cases diagnosed globally each year, and about 1.8 million patients succumbing to the disease [1, 2]. In global cancer mortality statistics, lung cancer’s fatality rate significantly exceeds that of other types. Pathologically, lung cancer is primarily categorized into small cell lung cancer (SCLC) and non-small cell lung cancer (NSCLC), with NSCLC accounting for roughly 85% of cases and SCLC about 15% [3].

In clinical diagnostics, the approaches for lung cancer diagnosis are primarily categorized into two major classes: imaging and pathology. Imaging diagnostic methods, particularly low-dose computed tomography (LDCT), are frequently utilized for early lung cancer screening due to their non-invasive nature. However, they are associated with a high rate of false positives, insufficient sensitivity, and concerns regarding radiation exposure. The National Lung Screening Trial (NLST) in the United States has demonstrated that the rate of false-positive results from low-dose computed tomography (LDCT) is as high as 96.4% [4]. These issues may represent significant barriers to the widespread adoption of early lung cancer screening, leading to delayed diagnoses for many patients [5, 6]. Optimized screening strategies, such as the integration of artificial intelligence (AI)-assisted imaging technology, radiomics, iterative reconstruction techniques, volumetric measurements, and risk assessment models, can reduce the risk of false-positive results and the likelihood of overdiagnosis [7]. Pathological histological diagnosis, considered the gold standard for lung cancer diagnosis, relies on invasive procedures such as biopsy or surgery to obtain tissue samples. The invasiveness, high costs, and potential for surgical complications pose substantial challenges, especially in developing and underdeveloped countries, representing a significant impediment for individuals at risk of developing tumors [8].

Surgical intervention remains the principal therapeutic modality for patients with lung cancer. For those deemed operable, the guiding principle as emphasized by clinical guidelines is the complete and radical resection of the tumor. In cases of partially resectable lung cancer, neoadjuvant chemotherapy and immunotherapy combined with chemotherapy are employed to downstage the tumor prior to surgical excision. However, for patients with lung cancer who are not candidates for surgical resection—this includes individuals with underlying conditions that preclude them from meeting surgical criteria or those with tumors in highly compromised locations—conventional radio-chemotherapy is typically the treatment of choice. Emerging minimally invasive techniques, such as Video-Assisted Thoracoscopic Surgery (VATS) and robotic-assisted surgery, not only minimize surgical trauma and subsequent complications but also have broad applicability in various traditional lung cancer resection procedures, facilitating more precise surgical excision [9]. The non-specific nature of chemotherapy, while combating the tumor, can also induce adverse effects such as myelosuppression, gastrointestinal reactions, and drug resistance, which contribute to a less than 30% efficacy rate in chemotherapy treatments. Radio-therapy, on the other hand, predominantly employs the method of stereotactic ablative radio-therapy (SABR). The cumulative toxicity of radiation and the hypoxic tumor microenvironment leads to radiotherapy resistance, further complicating the treatment landscape for lung cancer patients [10].

In recent years, the development of targeted therapies and immunotherapies has introduced new treatment modalities for lung cancer patients. In East Asia, the incidence of EGFR gene mutations (38–50%) is significantly higher than in America (24%) and Europe (14%), accounting for about 38% of adenocarcinomas [11–13]. In an international multicenter, open-label phase III clinical trial, despite an objective response rate of 83% with the combination of EGFR-targeted Osimertinib and chemotherapy, the 2-year disease-free survival rate was only 57% [14]. The application of immune checkpoint inhibitors targeting PD-1/PD-L1 and CTLA-4 has improved the clinical prognosis of NSCLC to some extent. However, the latest phase III clinical study (CheckMate 722, NCT02864251) showed that the use of nivolumab combined with chemotherapy after first-line treatment for EGFR-mutated metastatic NSCLC patients did not significantly improve progression-free survival (PFS: median, 5.6 months vs. 5.4 months) [15]. Lung cancer patients face key challenges on their path to survival, such as acquired mutations, bypass activation, histological transformation, off-target effects, complex changes in the tumor immune microenvironment, and the adaptive overexpression of various immunosuppressive receptors [16].

Since the 21st century, the rapid development of nanotechnology has shown great potential in the diagnosis and treatment of various malignant tumors [17, 18]. Nanoparticles, solid particles with sizes ranging from 1 to 100 nm, play a significant role in the field of tumor diagnosis and treatment due to their strong targeting and high modifiability [19]. For instance, a STING pathway activator (FeGd-HN@TA-Fe (2+)-SN38 nanoparticles) can be used for MRI-guided breast cancer immuno-ferroptosis synergistic therapy [20]. Additionally, a molecular imaging nanoprobe based on superparamagnetic iron oxide nanoparticles, using redox-active nitric oxide as a chemical target, quantifies the repolarization process from M2 to M1 macrophages via MRI [21]. In the field of tumor diagnosis and treatment, nanoparticles are increasingly used as nano-delivery systems, offering advantages such as high bioavailability, good solubility, strong in vivo stability, sustained delivery, and targeted delivery [19]. Nanocarriers deliver chemotherapeutic agents and natural products, enhancing cytotoxicity to tumor cells and preventing the development of drug resistance. Moreover, nano-delivery systems can also deliver CRISPR/Cas9, non-coding RNA, and RNAi, improving the efficiency of gene therapy and inhibiting tumor progression [22–24].

**Fig. 1:**
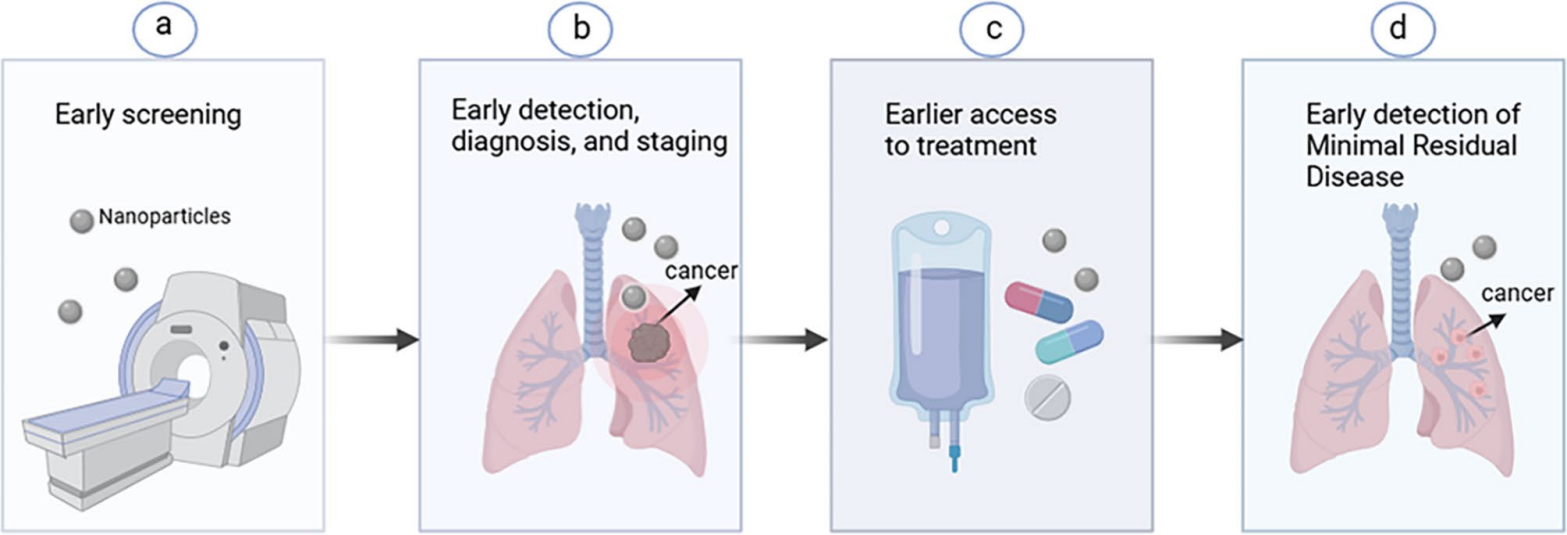
Nanotechnology plays a role at all stages of lung cancer diagnosis and treatment. **a**) Nanotechnology promotes early screening of lung cancer. **b**) Nanotechnology facilitates lung cancer diagnosis and staging. **c**) Nanotechnology promotes lung cancer therapy. **d**) Nanotechnology promotes the detection of minimal residual disease (MRD) in lung cancer

This review provides a comprehensive overview of the principal types of nanoparticles utilized in the field of lung cancer diagnosis and therapy. It encapsulates the research on nanoparticles within this domain, elucidating their applications in early screening, diagnostics, conventional treatments, targeted therapies, and immunotherapies for lung cancer. By critically assessing the strengths and limitations of various nanomaterials, along with their current application status and shortcomings throughout the entire spectrum of lung cancer management, this review highlights the burgeoning research value of nanomaterials in advancing lung cancer diagnostics and therapeutics (Fig. 1).

## Main types of nanomaterials used in lung cancer

### Lipids-based nanoparticles

The main types of nanomaterials applied in the field of lung cancer primarily include lipid-based nanocarriers. Lipid-based nanoparticles are one of the most common types of nanomedicines approved by the Food and Drug Administration (FDA). Traditional lipid nanoparticles are primarily composed of phospholipids, forming unilamellar or multilamellar vesicular structures, which enable them to deliver both hydrophilic and hydrophobic drugs, offering the advantages of high drug loading capacity and high biocompatibility. Lipid nanoparticles (LNPs), on the other hand, consist of cationic or ionizable lipids, phospholipids, cholesterol, and polyethylene glycol (PEG) lipids, forming a structure with a single-layer vesicle encapsulating a core micelle. Compared to traditional nanoparticles, LNPs have the advantages of smaller volume and higher serum stability [25, 26].

The components of LNPs determine their characteristics: cationic or ionizable lipids mainly couple with negatively charged genetic materials and aid in endosomal escape; phospholipids, as the main component of LNPs, are used to construct the particle structure; cholesterol maintains the stability of LNPs and promotes their fusion with the cell membrane; PEGylated lipids enhance the stability of LNPs, reduce immunogenicity, and extend their circulation time in the body [27].Lipid nanoparticles, including LNPs, possess the enhanced permeability and retention (EPR) effect, allowing them to accumulate in areas with increased vascular permeability, such as tumor sites, thus providing a significant advantage in tumor delivery. Despite the EPR effect being a cornerstone in the rationale for tumor-targeted drug delivery, it exhibits limitations in the clinical setting, particularly among patients with poorly vascularized tumors. For instance, elevated interstitial pressure within the tumor may restrict the permeation of drugs from vasculature to tumor tissue; insufficient lymphatic drainage could lead to a reduction in drug retention within the tumor; and the tumor stroma may form a physical barrier that impedes the penetration of drugs into the tumor tissue [28]. However, systemic cytotoxicity issues may still arise with LNP administration in both in vivo and clinical experiments [29]. The liver, due to its strong phagocytic and filtering functions, and the absence of an impermeable basement membrane, leads to a large accumulation of LNPs in the liver, which may not only cause liver toxicity and liver function damage but also affect the therapeutic effects of lung cancer.

In recent years, LNPs have become a research hotspot in the field of nano-oncology, with a multitude of preclinical and clinical studies based on LNPs emerging. To address the issue of excessive LNP accumulation in the liver, researchers have conducted a series of studies. For instance, Christoph et al. developed an LNP dry spray for siRNA drugs, aiming to overcome the challenge of LNPs primarily accumulating in the liver and having difficulty reaching the lungs. Studies have shown that this LNP dry spray can downregulate the expression of the housekeeping protein GAPDH in lung slices by 50%, proving the feasibility of LNP dry spray in delivering siRNA against lung cancer [30]. Researchers also utilized the characteristics of tumor-specific initiator factors/trans-acting factors (IF/ITAF) to identify the internal ribosome entry site (IRES) of the virus, encoding the IRES sequence of human rhinovirus type II (HRV2) before the GSDMD mRNA, thereby endowing the LNP drug GSDMDENG circRNA with the ability to target tumors, allowing more LNPs to accumulate in the tumor area and reducing systemic cytotoxicity [31]. LNPs are characterized by their high drug loading capacity, excellent biocompatibility, superior serum stability, and the ability to co-deliver both hydrophilic and hydrophobic drugs. Despite these advantages, LNPs also present certain drawbacks, including batch-to-batch variability, high manufacturing costs, and potential drug leakage [25].

### Bio-nanoparticles

Bio-nanoparticles in the field of lung cancer therapy are primarily categorized into several main classes: animal-derived proteins, antibody-drug conjugates (ADCs), viral nanoparticles and apoferritin.

### Protein-based nanoparticles

A 2021 review comprehensively discussed the clinical research and drug development status of protein-based nanomedicines from a pan-cancer perspective. This article focuses on the lung cancer field, supplementing preclinical research on protein nanoparticles and providing a detailed classification of several typical protein nanoparticles [32]. Albumin is the main animal-derived protein nanoparticle currently under study, mainly derived from egg white protein, bovine serum albumin (BSA), and human serum albumin (HSA). Albumin has excellent water solubility and the capacity to carry both water-soluble and insoluble drug molecules, with a surface that is easily modified and functionalized to overcome drug delivery barriers in cancer therapy [33, 34]. In recent years, research on albumin-based nano-delivery systems has emerged continuously, including studies on the delivery of natural anti-cancer drugs using albumin. For example, researchers have developed oleic acid (OLA) cetuximab (CTX) functionalized albumin nanoparticles (ALB-NPs) for EGFR-targeted lung cancer treatment, with in vitro and in vivo experiments confirming their strong targeting delivery capability and anti-tumor potential [35]. HSA possesses exceptional solubility for both hydrophilic and hydrophobic drug molecules, offering a robust drug-carrying capacity. Its surface is readily amenable to modification and functionalization, which can potentially surmount barriers encountered in drug delivery during anticancer therapy. However, the use of HSA as a drug carrier is not without limitations. These include variability in HSA, high costs associated with its production, limited availability of HSA sources, and minor immune responses upon administration of BSA [36]. Antibody-drug conjugates (ADCs) consist of three elements: monoclonal antibodies (MoAb), linkers, and potent drug payloads. ADCs deliver cytotoxic drugs selectively to tumor cells by targeting specific antigens expressed on the surface of cancer cells using monoclonal antibodies. The principle of ADCs is to utilize the Trojan horse mechanism of the drug to avoid cytotoxicity in non-tumor areas, precisely targeting the tumor area to kill cancer cells [37]. The targets of ADCs are mainly antigens expressed on the surface of lung cancer cells, including human epidermal growth factor receptor, trophoblast cell surface antigen, c-MET, carcinoembryonic antigen-related cell adhesion molecules, and B7-H3, among others [38]. Exatecan is an effective topoisomerase I inhibitor with low sensitivity to multidrug resistance (MDR). Researchers have designed ADCs primarily targeting HER2, HER3, and TROP2 antigens, conjugated to release exatecan. This drug has shown good therapeutic indicators, stability, and intra-tumoral pharmacodynamic response in lung cancer mouse models, with low drug toxicity, providing a new perspective for anti-lung cancer treatment [39]. ADCs integrates the advantages of antibody-specific targeting, prolonged circulation half-life, and the potency of highly cytotoxic anticancer agents. However, challenges such as treatment-related toxicity and therapeutic inefficacy due to tumor heterogeneity remain to be addressed.

### Viral nanoparticles

Viral nanoparticles (VNPs) are further divided into genetically engineered viruses and virus-like particles (VLPs), with adenoviruses (Ad) being representative of the genetically engineered category. Adenoviruses (Ad) are icosahedral non-enveloped viruses whose structural characteristics enable them to effectively encapsulate and deliver anti-cancer drugs. However, systemic administration of naked adenoviruses may induce severe hepatotoxicity and trigger rapid immune responses in the host due to their highly immunogenic viral capsids, leading to severe inflammatory reactions [32, 40]. To address these issues, researchers have developed a polyethylene glycol (PEG)-modified PAMAM (PPE) complexed oncolytic adenovirus co-expressing decorin (DCN) and c-Met-specific short hairpin RNA (shMet), which reduces the high immunogenicity of adenoviruses. By conjugating EGFR-specific antibodies (ErbB) on the surface of PPE, leveraging the specific expression of EGFR on lung cancer cells, the enrichment rate of adenovirus nanoparticles in the lung cancer area is maximized, reducing hepatotoxicity caused by systemic administration. Additionally, by downregulating the c-Met signaling pathway, tumor cell growth is inhibited, and tumor cell autophagy is induced, significantly improving the therapeutic effect on lung cancer [41]. Researchers have also encapsulated adenovirus nanoparticles with tumor cell membranes, creating ExtraCRAd nanoparticles, which utilize tumor-associated antigens on the tumor cell membrane to achieve greater tumor targeting and hepatotoxicity avoidance. Both in vitro and in vivo experiments have confirmed their potent oncolytic effects and inhibitory effects on lung cancer growth [42]. Ad possess advantages such as high transduction efficiency, large drug payloads, and scalability. However, their capsid’s immunogenicity triggers innate and adaptive immune responses, hindering systemic nanoparticle administration. Clinically, intravenous adenoviral nanoparticles are swiftly neutralized and eliminated, triggering inflammation and adverse effects through host antiviral immunity activation. To ameliorate these issues, approaches include genetic modification to ablate immunogenic genes, immunoadjuvant incorporation to boost tumor-specific immunity, and combination with immunotherapies (e.g., checkpoint inhibitors) to enhance antitumor responses, thereby modulating immune reactions and reducing clinical side effects [43].

### Apoferritin

Ferritin, an iron-storage protein ubiquitous in biological systems, plays a pivotal role in iron metabolism and cellular antioxidant maintenance. Apoferritin, the iron-depleted form of ferritin, is composed of 24 self-assembled subunits that form a spherical nanocage with a diameter of approximately 12 nm, featuring an internal cavity of 8 nm capable of encapsulating drugs, thereby constituting a ferritin drug carrier (FDC). Protein C nanoparticles (PCNs) based on ferritin, termed TFG and TFMG, significantly inhibit hypoxia and enhance pericyte coverage, thereby suppressing tumor growth and metastasis. Moreover, PCNs alter the immune cell profile within tumors by increasing cytotoxic T cells with antitumor activity and M1-like macrophages, offering effective therapeutic strategies [44]. Human ferritin nanocages (FTn) possess an inherent ability to preferentially target hypoxic tumor tissues. Huang et al. endowed FTn with spatially controlled “mosaic” surface polyethylene glycol (PEG) coatings, facilitating FTn’s deep penetration through the tumor extracellular matrix (ECM) to reach hypoxic tumor tissues, providing significant therapeutic benefits [45]. FDCs exhibit superior surface modifiability, drug loading capacity, thermal stability, solubility, abundance in blood, and low toxicity [46]. Research on the anticancer potential of FDCs remains challenging, as the drug loading capacity and distribution of drugs within FDCs may affect their efficacy and stability, and clinical studies validating their anticancer potential are still lacking [47].

### Polymeric nanoparticles

Polymeric Nanoparticles (PNPs) are a class of solid or colloidal nanoscale carriers with sizes ranging from approximately 10–1000 nm. PNPs mainly include forms such as nanospheres, nanocapsules, and nanomicelles. Nanospheres are solid spherical nanoparticles that can couple drug molecules on their surface or encapsulate drugs within the particle; nanocapsules have a solid shell and a liquid cavity for drug loading. As drug carriers, PNPs exhibit high thermodynamic stability. Their small size helps avoid renal clearance and demonstrates good endothelial permeability, making PNPs significantly promising in the field of cancer therapy. Hydrophobic drugs can be covalently bound to PNPs and effectively encapsulated in the core of PNPs. PNPs can be administered through various routes, including injection and oral administration. Despite challenges in targeting, cellular drug resistance, and systemic toxicity from administration, PNPs offer a highly promising new strategy as nanomedicine delivery vehicles in the diagnosis and treatment of lung cancer [48–52].For instance, biomimetic nanoparticles loaded with EGFR-TKI-Osimertinib (CMNP(@Osi)), featuring a polymeric nanoparticle core and coated with tumor cell membranes, utilize antigens on the tumor cell membrane and molecularly targeted drugs to achieve dual-targeting [53]. To address the issue of cytotoxicity, the delivery of bevacizumab mRNA via poly (beta-amino ester) (PBAE) nanoparticles targeted to the lungs has demonstrated a more pronounced inhibitory effect on tumor proliferation and angiogenesis in a murine model of orthotopic non-small cell lung cancer (NSCLC). This approach effectively circumvents the adverse systemic effects associated with the systemic administration of anti-VEGF antibodies in the treatment of NSCLC [54]. PNPs serve as promising drug carriers due to their high thermodynamic stability. Their nanoscale dimensions facilitate evasion of renal clearance and enhance permeability across endothelial cells. Additionally, PNPs offer several advantages, including facile synthesis, cost-effectiveness, biocompatibility, non-immunogenicity, non-toxicity, and aqueous solubility, which collectively render them highly promising in the field of cancer therapeutics. However, there are concerns regarding potential particle aggregation and toxicity associated with PNPs [55].

### Metal nanoparticles

Metal nanoparticles, derived from metal compounds such as metal oxides and metal salts, can be categorized into pure metals, metal oxides, and metal chalcogenides. Gold (Au), silver (Ag), iron (Fe), copper (Cu), and platinum (Pt) are the primary elements that constitute metal nanoparticles. With their unique physicochemical properties—magnetic, optical, thermal, and electrochemical—metal nanoparticles have found extensive applications in the diagnosis and treatment of lung cancer. These applications include imaging enhancement, biosensors, cancer biomarker detection, drug delivery reinforcement, chemo-sensitization, and combination with photodynamic therapy. Zheng and colleagues summarized the advanced metal nanoparticles applied in lung cancer therapy and diagnosis in recent years in 2023 and discussed the future development of metal nanomedicine. Therefore, this article will focus on the research progress of metal nanoparticles in the field of lung cancer post-2023 [56].

A dual-quenched electrochemiluminescence resonance energy transfer (ECL-RET) immunosensor based on iron-based metal-organic frameworks (FeMOFs) loaded with small-sized copper oxide (CuO) nanoparticles (FeMOFs-sCuO) transferring to cobalt-palladium (CoPd) nanoparticles (CoPdNPs) has demonstrated exceptional sensitivity, specificity, and reproducibility. This sensor supports highly sensitive analysis of neuron-specific enolase (NSE) and shows potential application value in the clinical diagnostic analysis of small cell lung cancer (SCLC) [57]. Additionally, a novel liquid biopsy method for non-small cell lung cancer (NSCLC), laser desorption ionization time-of-flight mass spectrometry (LDI-TOF MS), utilizes gold nanoparticles/cellulose nanocrystals (AuNPs/CNC) as a matrix to directly analyze intact proteins in serum exosomes. This method improves the ion suppression effect caused by protein aggregation, significantly enhancing the sensitivity of LDI-TOF MS technology [58]. Metal nanoparticles can also be coupled or modified with other materials to form multifunctional metal nanoparticles, further expanding their application scope in lung tumor diagnosis and treatment [59–61]. For example, superparamagnetic iron oxide nanoparticles (SPION-CCPMs) modified with cross-linked polymer micelles can restructure the immunosuppressive pulmonary tumor microenvironment (TME), enhance the recruitment and cytotoxic characteristics of CD8 + T cells, and promote the transition of tumor-associated macrophages (TAMs) to a pro-inflammatory phenotype, exerting antitumor activity through the secretion of reactive nitrogen species and cytokines. These micelles, connected via disulfide bonds, augment the stability of nanoparticles and render the release of SPION-CCPMs dependent on intracellular glutathione (GSH) within macrophage endosomes, ensuring effective iron payload within macrophages [62, 63]. Metal-organic framework (MOF) nanoparticles loaded with polyphenols and peroxymonosulfate (PMS, ZIF-67@ZIF-90) adhere to the surface of macrophages (Mφ), constructing “macrophage missiles” PMS@ZIF-67@ZIF-90@Mφ. These Mφ can selectively recognize and target tumor cells, accurately delivering nanomedicines to the lesion, thereby reducing cytotoxicity and systemic side effects. PMS demonstrates higher efficacy and lower toxicity under oxygen-independent conditions [64]. Given the widespread risk of postoperative bacterial pneumonia in lung cancer patients, which significantly impacts treatment and prognosis, research into metal nanomedicines with synergistic antibacterial and anti-lung cancer properties has become increasingly relevant [60, 65]. Microbial capsule polysaccharide (CP) coated gallium-polyphenol metal-organic networks (MON), namely GaTa-CP NP, use Ga (3+) to disrupt bacterial iron respiration to eliminate microbes, while CP, by mimicking normal host tissue, reduces immune clearance rates and prolongs the residence time of nanoparticles in lung tissue, thereby enhancing antibacterial capabilities and eliminating microbial-induced chemoresistance [66].

**Fig. 2:**
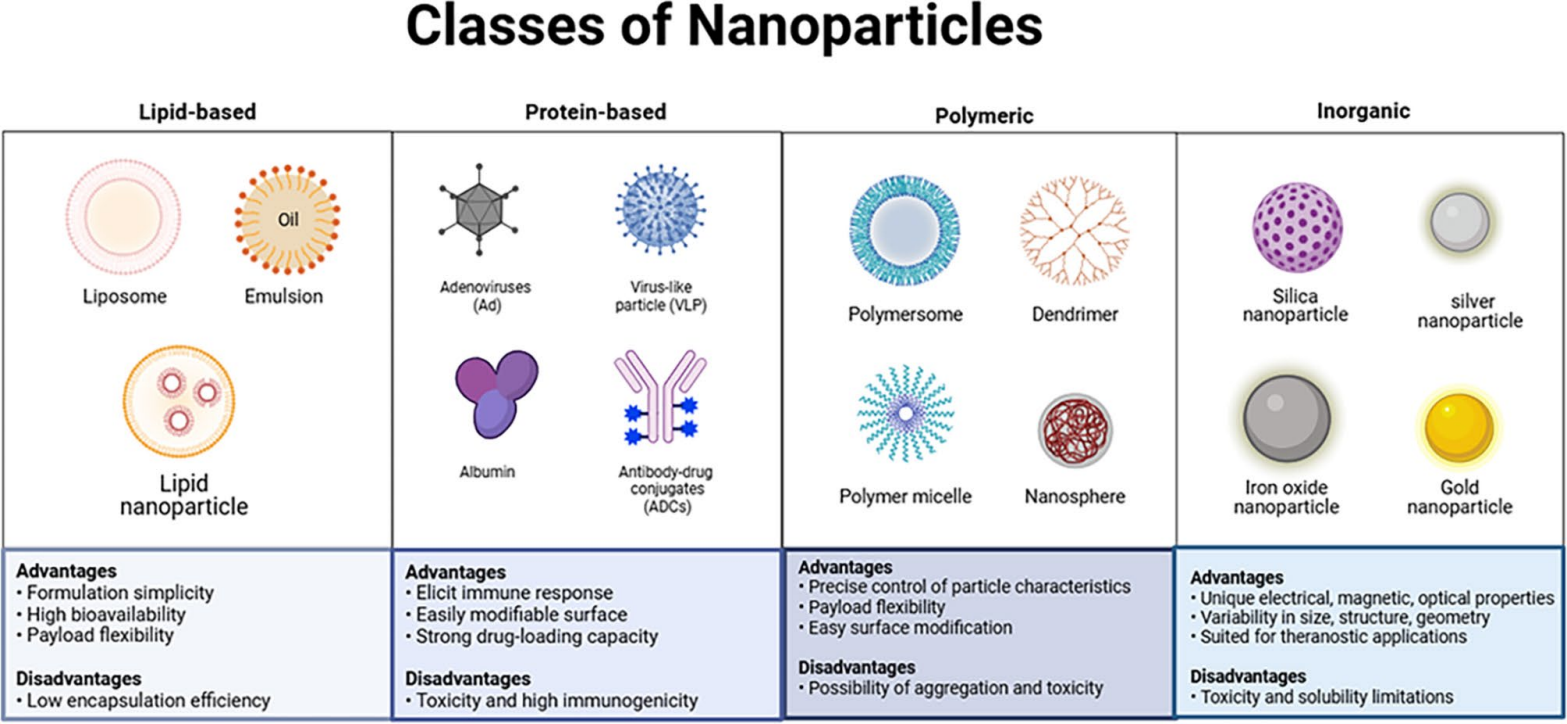
Main nanomaterials of lung cancer. Here, the main subclasses of lipid-based nanomaterials, protein-based nanomaterials, polymeric nanomaterials, and metallic nanomaterials are introduced, along with their main advantages and disadvantages

These research advancements indicate that different types of nanomaterials, leveraging their inherent strengths, hold great potential in improving drug delivery efficiency, enhancing therapeutic effects, and reducing treatment side effects, providing new insights and strategies for precision therapy in lung cancer. Metal nanoparticles have garnered widespread interest in lung cancer research due to their facile synthesis, functionalization capabilities, high biocompatibility, and multifaceted theranostics profile。However, they are not without drawbacks, such as low endosomal escape efficiency, hemolytic activity, high nephrotoxicity, and short circulation times [67, 68] (Table 1) (Fig. 2).

**Table 1:** Main nanoparticles applied in the field of lung cancer and their advantages and disadvantages

Nanoparticles type	Advantages	Disadvantages	Representative example
Lipids-based nanoparticles	Deliver both hydrophilic and hydrophobic drugs, high drug loading capacity, high biocompatibility and superior serum stability	Batch-to-batch variability, high manufacturing costs, potential drug leakage, systemic cytotoxicity, liver toxicity	GSDMD^*ENG*^ circRNA
Protein-based nanoparticles	Easily modifiable and functionalizable, deliver both hydrophilic and hydrophobic drugs	Albumin variability, high costs, limited sources of HAS and immune responses of BSA	ALB-NPs
Viral Nanoparticles	High delivery efficiency, large drug loading capacity and scalable production	High immunogenicity, liver toxicity	PPE-Ad
Apoferritin	Superior surface modifiability, drug loading capacity, thermal stability, solubility, abundance in blood and low toxicity	Drug loading capacity can impact its therapeutic efficacy and stability	FTn
Polymeric Nanoparticles	High thermodynamic stability, biodegradability, good endothelial cell permeability, facile synthesis, cost-effectiveness, biocompatibility, non-immunogenicity, non-toxicity and aqueous solubility	Particle aggregation, toxicity, poor targeting	CMNP(@Osi)
Metal nanoparticles	Facile synthesis, functionalization capabilities, high biocompatibility and multifaceted theranostics profile	Low endosomal escape efficiency, hemolytic activity, High nephrotoxicity	SPION-CCPMs

a)**GSDMD**^***ENG***^**circRNA**,engineered HRV2 IRESs-GSDMD^*p.D275E/E295G*^-F1L^CT^ circRNA-LNPs; b)**ALB-NPs**,albumin nanoparticles; c)**PPE-Ad**,epidermal growth factor receptor-specific therapeutic antibody-conjugated and PEGylated poly(amidoamine) dendrimer (PPE) for complexation with Ad; d)**FTn**,human ferritin nanocages; e)**CMNP(@Osi)**,biomimetic nanoparticles loaded with EGFR-TKI-Osimertinib; f)**SPION-CCPMs**,superparamagnetic iron oxide nanoparticles; g)**HAS**,human serum albumin; h)**BSA**,bovine serum albumin

## Research progress of nanomaterials in detection and diagnosis of lung cancer

### Research progress of nanomaterials in detection of lung cancer

#### Imaging examination

The early detection of lung cancer is closely associated with treatment outcomes and prognosis. However, the current rate of early screening for lung cancer is not satisfactory, with many patients diagnosed at an advanced stage. This phenomenon is often related to the limitations of conventional lung cancer screening methods. Imaging examinations, such as low-dose computed tomography (LDCT), are the most direct means of early screening, but they are limited by a high rate of false positives, significant radiation exposure, and insufficient sensitivity to detect small lesions, thereby constraining the effectiveness of early screening. A phase I/II clinical study (NCT04789486) indicated that a gadolinium-based nanoparticle (AGuIX), used as a contrast agent for stereotactic magnetic resonance (MR) imaging, may enhance tumor radiosensitivity and improve the clarity with which physicians visualize tumors. However, clinical research on the application of nanoparticles for early lung cancer screening is lacking. Numerous preclinical studies have indicated that nanomaterials, used as contrast agents or nano-probes, can enhance the sensitivity of clinical imaging, potentially aiding in the tracking of small lesions and early screening [69]. In particular, nanomaterials targeted to the location of lung cancer, utilizing their physicochemical properties or the fluorescent molecules they carry, can enable visualization of the cancer site and provide real-time monitoring of lung cancer and its metastatic pathways. For instance, intratracheal administration of perfluoro-15-crown-5-ether nanoparticles (PFCE NPs), transported to the tumor periphery via the phagocytic action of pulmonary macrophages, increases the concentration of PFCE NPs in the tumor area and expresses [19]F-MRI signals, effectively achieving visualization of lung cancer [70].The insensitivity of magnetic resonance imaging (MRI) and the low efficiency of ultrasound energy deposition have limited the application of magnetic resonance-guided focused ultrasound surgery (MRgFUS) in non-invasive cancer treatment. Polyethylene glycol (PEG) SPIO nanoparticles, surface-modified with anti-EGFR antibodies and targeted to EGFR-overexpressing lung cancer, not only enhance MRI contrast at the tumor site but also significantly improve the efficiency of ultrasound energy deposition, promoting the development of MRgFUS [71]. MRI and ultra-small rigid platforms (USRP) serve as powerful imaging tools capable of non-invasively detecting, quantifying, and longitudinally monitoring the progression of sub-millimeter NSCLC [72]. The deposition of USRP around tumor nodules after nebulization produces a radiosensitizing effect, and the non-invasive detection of lung tumors through fluorescence tomography or UTE-MRI, without observed systemic toxicity or inflammation induction during the experimental process, demonstrates its advantages in multimodal detection of lung tumors and improving the efficacy of radio-therapy [73].

#### Biosensor

In traditional lung cancer diagnosis, solid biopsy and liquid biopsy are key detection methods. Solid biopsy, an invasive method to obtain tumor tissue samples, is more harmful to patients, especially unsuitable for those in advanced stages. Liquid biopsy, as a non-invasive detection method, involves collecting peripheral blood and body fluids for tumor marker detection but requires a large volume of blood samples, with high detection costs and the need for improved accuracy and sensitivity. Nanomaterials, as biosensors, offer a non-invasive, simple sampling, and highly sensitive detection method, overcoming the invasiveness of solid biopsy and the large sample volume requirement of liquid biopsy. Biosensors can simultaneously detect multiple tumor markers, improving detection efficiency and the accuracy of tumor diagnosis [74, 75]. Specifically, sensor arrays based on gold nanoparticles can rapidly differentiate the breath of lung cancer patients from healthy individuals in high-humidity environments [76]. Coupled with solid-phase microextraction and gas chromatography or mass spectrometry, they can identify volatile organic compounds in lung cancer biomarkers, potentially serving as an inexpensive and non-invasive diagnostic tool for lung cancer. Nanomaterials such as Ni-OTC NPs and PdCuB MNs, with their exceptional catalytic activity, have significantly enhanced the sensitivity of biosensors in detecting low-abundance biomarkers, such as EGFR exon 21 mutations and PD-L1 exosomes. Furthermore, through electropolymerization and functional modification, these nanomaterials have optimized the electron transfer efficiency of the sensors, achieving rapid response. Nanocomposite materials like rGO/f-OMC and NG-THI-AuNPs, with their specific chemical and physical properties, endow the sensors with high selectivity, effectively reducing the risk of cross-reactivity and false positives. This contributes to the improvement of accuracy and timeliness in clinical treatment, providing lung cancer patients with more precise diagnostic tools. The incorporation of nanomaterials not only enhances the stability and durability of biosensors, as demonstrated by the 21-day stability of electrochemical gene biosensors, but also, through their high surface area and surface effects, reduces the detection limit to 120 nM, thereby increasing the sensitivity of detection [77–80].

#### Theragnosis integration

To achieve the high efficiency of lung cancer treatment, the design of nanomaterials often focuses on the dual functions of real-time imaging and therapy, elevating the integration of diagnosis and treatment to a new level. The theranostic nano-platform, which combines upconversion nanoparticles (UCNPs) with IR-1048 dye in lipidic aptamer nanostructures (UCILA), not only exhibits superior luminescent properties and a high X-ray attenuation coefficient but also enables real-time monitoring of tumor metabolic activity and temperature changes, providing feedback on the efficacy of photothermal therapy (PTT). The synergistic effect of this platform not only promotes the integration of photothermal therapy and tumor-targeted immunotherapy but also offers an intuitive approach to obtaining comprehensive structural and functional information of lung cancer [81]. The preparation and characterization of the Mn-porphyrin & Fe3O4@SiO2 nanocomposite material, further modified with @PAA-cRGD, have successfully achieved its application in T1- and T2-weighted MRI as well as pH-responsive drug release. Fluorescence imaging results indicate that this nanocomposite material can specifically accumulate in lung cancer cells and target the treatment of lung cancer cells, while showing no toxicity to normal cells. By integrating imaging diagnostics with controlled drug release, this nanocomposite material provides real-time imaging during targeted therapy, significantly enhancing the accuracy of diagnosis and demonstrating its broad application prospects in lung cancer treatment [82].

**Fig. 3:**
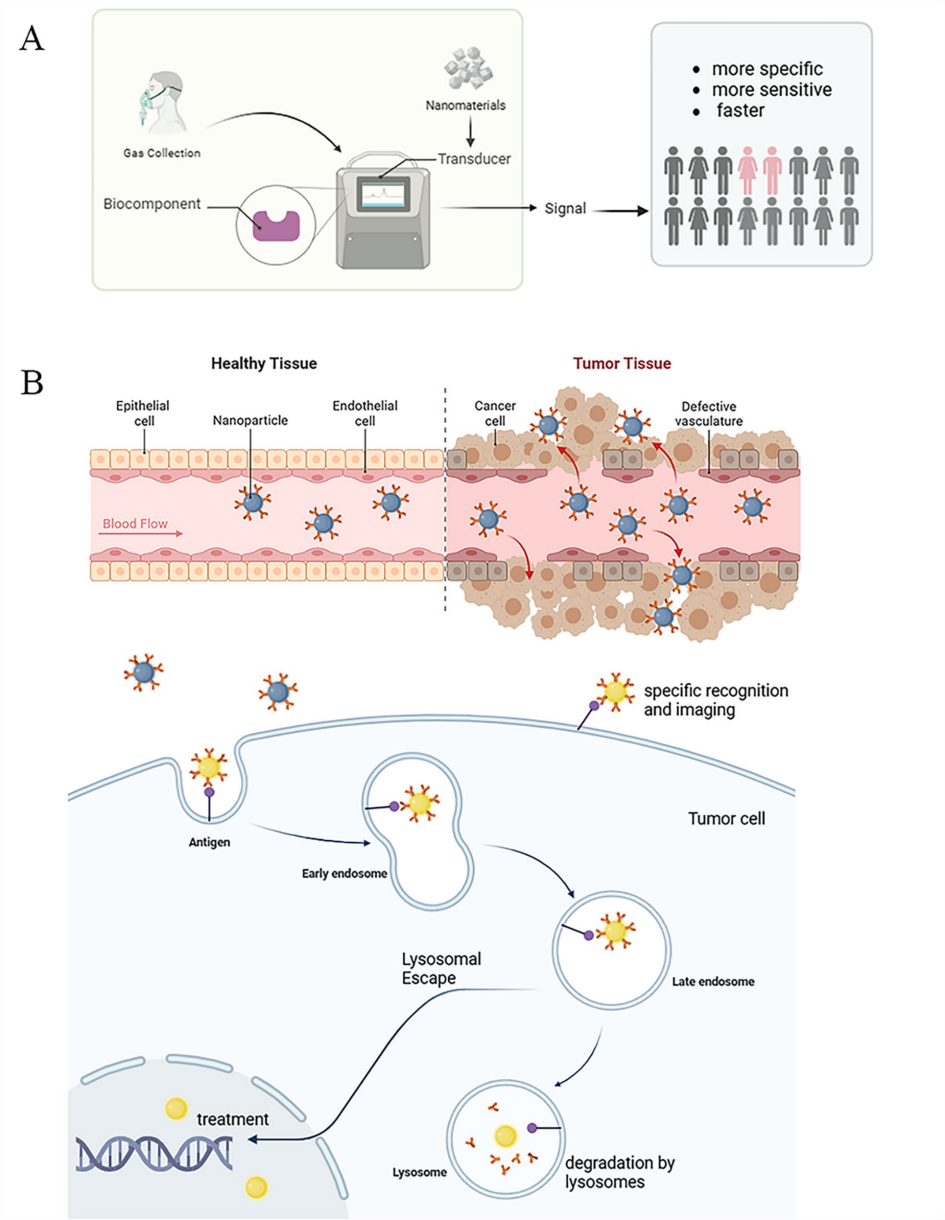
Research and application of nanotechnology in lung Cancer Diagnosis. **A**) Non-invasive screening of lung cancer patients using nanotechnology combined with biosensors; **B**) Nanotechnology, serving as a contrast agent or nano-probe, not only specifically recognizes lung cancer cells to perform imaging functions but also possesses therapeutic effects

In addition, nanotechnology such as nano-gold labeling techniques, nanomaterial-assisted PCR techniques, nanopore sequencing techniques, and bio-barcoding techniques, provide new tools for the in vitro detection and analysis of proteins or nucleic acids, potentially paving new avenues for molecular diagnosis of lung cancer. In summary, nanotechnology demonstrates immense potential in the early screening and diagnosis of lung cancer (Fig. 3).

### Research progress of nanomaterials in therapy of lung cancer

#### Research progress of nanomaterials in surgical treatment of lung cancer

In the field of lung cancer treatment, surgical resection, radiation therapy, and chemotherapy constitute traditional therapeutic approaches. Surgical resection, whether for curative or palliative purposes, remains the first choice of treatment. Studies have indicated that nanotheranostic H points, loaded with gefitinib and quercetin, can serve dual roles in lung cancer surgery by providing image-guided navigation and drug delivery. These nanotheranostic agents facilitate the precise determination of surgical margins through imaging guidance, enabling accurate resection, while also delivering anticancer drugs to exert a synergistic therapeutic effect [83]. Furthermore, the advent of nanorobots signifies the potential for a non-invasive surgical era in the near future. Addressing the challenges associated with the clinical translation of traditional nanocarriers, more powerful and proactive tumor-targeting systems—nanorobots—have been developed. These nanorobots are capable of assisting surgeons in performing more precise and complex procedures, effectively clearing tumor margins [84, 85]. The first generation of DNA nanorobots integrates biological and mechanical systems, administered via intravenous injection to perform non-invasive endovascular interventional surgery, accurately delivering thrombin to produce thrombosis, cutting off the blood supply to the tumor, effectively inhibiting the growth and metastasis of lung cancer [86].

#### Research progress of nanomaterials in radio-therapy of lung cancer

Radio-therapy works by damaging the chromosomes of cancer cells to inhibit their proliferation. However, hypoxic conditions in the tumor microenvironment and the characteristics of cancer stem cells (CSCs) severely limit the efficacy of radio-therapy [87].

Under hypoxic conditions, tumor cells significantly increase their resistance to radio-therapy and promote tumor angiogenesis, protecting endothelial cells within blood vessels and resisting the cytotoxic effects of radio-therapy. Nanoparticles can overcome the inhibition of radio-therapy by hypoxic environments and enhance the toxicity of radio-therapy to tumor tissues [88]. For example, self-assembled pH-sensitive superparamagnetic iron oxide nanoclusters (SPIONCs) release iron ions in an acidic microenvironment, generating hydroxyl radicals under X-ray irradiation, inducing apoptosis in lung cancer cells [89]. Cancer stem cells possess the ability to self-renew and differentiate, and they can resist radio-therapy through various pathways [90]. To improve the effectiveness of radio-therapy, radiosensitizers are commonly used during radio-therapy. Nanomaterials based on high atomic number elements, such as gold, silver, bismuth, and gadolinium, serve as radiosensitizers, effectively concentrating more radiation energy in the tumor area while reducing damage to normal tissues [91]. AGuIX nanoparticles (AGuIX-Bi) combine a ratio of 30Gd/70Bi, providing maximum safety and efficacy while amplifying the radiation dose [92]. Photothermal therapy disperses pH/laser-triggered clustered nano-systems into ultra-small gold nanoclusters, enhancing tumor penetration ability and radio-sensitization effects [93].

#### Research progress of nanomaterials in chemotherapy of lung cancer

Chemotherapy is a therapeutic approach that uses chemical agents to arrest the cell cycle and promote apoptosis to kill cancer cells. It has the advantages of rapid onset, significant effects, and a broad range of action. However, it also comes with significant cytotoxicity and the issue of drug resistance.

The research of nanotechnology in chemotherapy can address the cytotoxicity of chemotherapy and enhance therapeutic outcomes. Nano delivery systems can accurately deliver drugs to the tumor site, protect chemotherapeutic agents from degradation during delivery, and reduce harm to normal tissues. For example, methoxy-PEG and gallic acid complexed cisplatin nanoparticles (PEG-GAx/Pt) rapidly dissociate and release the drug in response to tumor acidity or elevated levels of reactive oxygen species, improving antitumor efficiency and reducing toxicity [94]. Albumin-encapsulated Pt (IV) nanomedicine (HSA@Pt(IV)) can activate ferroptosis without affecting normal tissues [95]. Carboxymethyl chitosan-modified nanoparticles (T7-CMCS-BAPE, CBT) can target lung cancer cells and precisely regulate drug release, enhancing biosafety and antitumor effects [96]. Intratumoral heterogeneity in cellular composition, molecular expression, microenvironment, and therapeutic response may influence the efficacy of nanoparticles. Therefore, enhancing the targeted design, stimulus-responsive design, multifunctionality, and optimizing the size and shape of nanoparticles may mitigate the impact of tumor heterogeneity on the therapeutic efficacy of nanomedicines [97]. The development of chemotherapeutic drug resistance is associated with factors such as tumor heterogeneity, overexpression of ABC transporters, epithelial-mesenchymal transition, and genetic mutations. Nanoparticles can assist in reversing chemotherapeutic drug resistance [98]. For instance, Fluplatin@PEG-PE nanoparticles (FP NPs) can degrade mutp53 and trigger endoplasmic reticulum stress, alleviating resistance to cisplatin chemotherapy [99]. DA-P-SS-T/PTX nano-micelles rapidly release drugs at high GSH levels, leading to apoptosis in drug-resistant lung cancer cells [100]. CNT-DOX nanomedicine, through covalent bonding of doxorubicin with carbon nanotubes of specific nanoscale dimensions, stably resides within drug-resistant tumor cells for an extended period, effectively treating drug-resistant lung cancer [101].The issue of chemotherapeutic drug insensitivity also affects treatment efficacy. Nano delivery systems co-delivering multiple drugs can enhance chemosensitivity and antitumor effects. The CLip-PC@CO-LC NPs delivery system co-delivers siPGAM1 and DTX, enhancing the chemosensitivity and antitumor capability of lung cancer [102].

The combined use of radio-therapy and chemotherapy, supplemented by nanotechnology, can improve therapeutic outcomes and reduce side effects. X-ray responsive peptide nanogels (PNG) are used to deliver chemotherapy drugs triggered by radio-therapy, synergistically enhancing the efficacy of radio-chemotherapy. Smart PNG accelerates drug release under X-ray irradiation, showing better synergistic antitumor efficacy and fewer side effects when combined with X-ray irradiation [103].

Nanotechnology has demonstrated immense potential in the traditional treatment of lung cancer, providing new insights and strategies for the precise treatment of lung cancer.

#### Research progress of nanomaterials in targeted therapy of lung cancer

Targeted therapy for lung cancer involves the use of drugs that act on specific genes, proteins, or other molecular markers present in tumor cells. This therapeutic strategy leverages the differences between tumor and normal cells to precisely target the medication to tumor cells, effectively killing or inhibiting tumor cells while minimizing the impact on normal tissues. Current targeted drugs for lung cancer mainly include anti-angiogenesis drugs, epidermal growth factor receptor (EGFR) inhibitors, and anaplastic lymphoma kinase (ALK) positive inhibitors. Specific medications include bevacizumab for anti-angiogenesis, gefitinib and osimertinib for EGFR, and crizotinib for ALK-positive patients. However, targeted drugs often target receptors that are highly expressed in tumors and lowly or not expressed in normal tissues, leading to certain side effects [104, 105].

The use of nano delivery systems can achieve dual targeting of drugs, reduce damage to normal tissues, and increase drug concentration in the tumor area. For example, hyaluronic acid (HA) modified pH-sensitive lipid polymer hybrid nanoparticles (LPH NPs) co-deliver erlotinib (ERL) and bevacizumab (BEV), forming HA-ERL/BEV-LPH NPs that exhibit high tumor tissue accumulation and low systemic toxicity. Bevacizumab is coated on the surface of gefitinib-loaded nanoparticles (BCGN) through electrostatic action, effectively accumulating in NSCLC tumors after intravenous injection and releasing in the tumor microenvironment, significantly inhibiting lung tumor growth. Additionally, red blood cell membrane-coated albumin nanoparticles (R-RBC@GEF-NPs) developed by Qian et al., enhanced tumor targeting with cRGD peptides, showed inhibition of A549 cell growth by inducing G1 phase apoptosis and cycle arrest in vitro, and real-time tumor imaging was achieved through Tc labeling in vivo [106–108].

Despite the good efficacy of targeted drugs, the occurrence of drug resistance remains an inevitable challenge. Nanotechnology has shown great potential in overcoming drug resistance to targeted drugs. For instance, CuS nanoparticles act as a photodynamic nano-switch, specifically eliminating bypass signaling in drug-resistant tumor cells and enhancing the tumor’s sensitivity to gefitinib treatment. Moreover, block copolymer dendrimer nanoparticles co-delivering EGFR-TKI gefitinib and YAP-siRNA successfully achieve lysosomal escape and responsive drug release, significantly overcoming drug resistance in NSCLC cells [109, 110].

Osimertinib (OSI), as a third-generation EGFR tyrosine kinase inhibitor (TKI), and the combined therapy with selumetinib (SEL) is a promising strategy to overcome drug resistance. SEL is conjugated with PEG using a reactive oxygen species (ROS)-responsive linker, forming a PEG-SEL prodrug that self-assembles into micelles as a delivery vehicle for OSI, co-delivering OSI and SEL nanoparticles effectively overcoming OSI resistance in NSCLC [111].For ALK-positive NSCLC patients, although there is a good response to ALK tyrosine kinase inhibitor (TKI) therapy, drug resistance eventually develops. Superparamagnetic iron oxide nanoparticles (SPION-CCPM) stimulate tumor-associated macrophages (TAM) to secrete reactive nitrogen and cytokines, reshaping the immunosuppressive lung tumor microenvironment, delaying tumor growth, preventing the recurrence of tumors, and proving as an adjunct therapy to delay lung cancer cell resistance [62].

The research of nanotechnology in targeted therapy not only solves the problem of drug resistance and enhances therapeutic effects but also enhances the sensitivity of targeted drugs. For example, bevacizumab (Bev), as the first-generation angiogenesis inhibitor, has become the standard first-line treatment for advanced non-small cell lung cancer (NSCLC). Polyphenolic compounds isolated from propolis (PCIBP) encapsulated in hybrid peptide-protein hydrogel nanoparticles (EPCIBP), when used in combination with Bev, can enhance the effectiveness of Bev and reduce the required dosage [112].

In summary, nanotechnology has improved the targeting and sensitivity of lung cancer targeted drugs and has shown great potential in overcoming drug resistance.

#### Research progress of nanomaterials in immunotherapy of lung cancer

Immunotherapy stands as a pivotal cancer treatment modality, alongside surgery, radio-therapy, and chemotherapy, demonstrating significant success rates in clinical applications. However, the efficacy of immunotherapeutic agents is often hindered by the immunosuppressive tumor microenvironment, which impedes effective penetration and leads to rapid metabolism and short residence times at the tumor site, resulting in insufficient activation of immune responses and limited therapeutic outcomes. The application of nanotechnology offers new hope in addressing these challenges [113, 114].

The rapid metabolism of single immunoadjuvant drugs makes it difficult for them to accumulate at the tumor site, affecting their clinical efficacy. Immunogenic cell death (ICD), as a novel antitumor strategy, provides tumor-associated antigens that activate dendritic cells and attract lymphocyte-like T cells into the tumor microenvironment. For instance, doxorubicin-induced tumor membrane-coated iron(II)-cytosine-phosphate-guanine nanoparticles (DM@NPs) achieve effective co-delivery of tumor-associated antigens and adjuvants, promoting dendritic cell maturation and the release of pro-inflammatory cytokines, significantly increasing T-cell infiltration, reshaping the antitumor immune microenvironment, and inhibiting the progression of lung cancer [115]. Low drug delivery efficiency increases medication costs and reduces therapeutic effects. Self-assembled lipid bilayer nanoparticles loaded with podophyllotoxin not only reduce the systemic toxicity of the drug but also enhance its penetration into tumors, improving antitumor efficacy and suppressing the production of programmed death-ligand 1 in lung cancer cells, promoting tumor-specific immune responses and providing strategies for modulating the immunosuppressive lung cancer microenvironment [116]. mRNA-based cancer immunotherapies often utilize lipid nanoparticles (LNPs) for effective delivery of mRNA payloads. In lung cancer cells, specific LNP formulations significantly enhance the delivery efficiency of circular RNAs (circRNAs) and effectively activate immune responses. A single intra-tumoral injection of LNPs loaded with circRNA encoding interleukin-12 (IL-12) induced a robust immune response in lung cancer models, leading to significant tumor regression [117].

Immune checkpoint inhibitors (ICIs) targeting PD-L1 and PD-1 have improved the survival rates of patients with advanced non-small cell lung cancer (NSCLC). The therapeutic effect depends on the degree of immune cell infiltration within the tumor tissue, with cancers classified as “hot tumors” or “cold tumors” based on immune cell infiltration in the tumor microenvironment. “Hot tumors” respond well to ICIs, while “cold tumors,” lacking immune cell infiltration, show poor responses. In lung cancer, only a minority of NSCLC patients respond to ICIs, particularly those with spinal metastases (NSCLC-SM) who are insensitive to ICIs. The application of nanotechnology offers new hope for these patients. Studies have shown that integrin β3 (β3-int) inhibitor RGDyK-modified mesoporous silica nanoparticles (ZnPP@MSN-RGDyK) can accurately target β3-int, inhibit PD-L1, and exhibit excellent immune therapeutic effects in the NSCLC-SM mouse model [118].

**Fig. 4:**
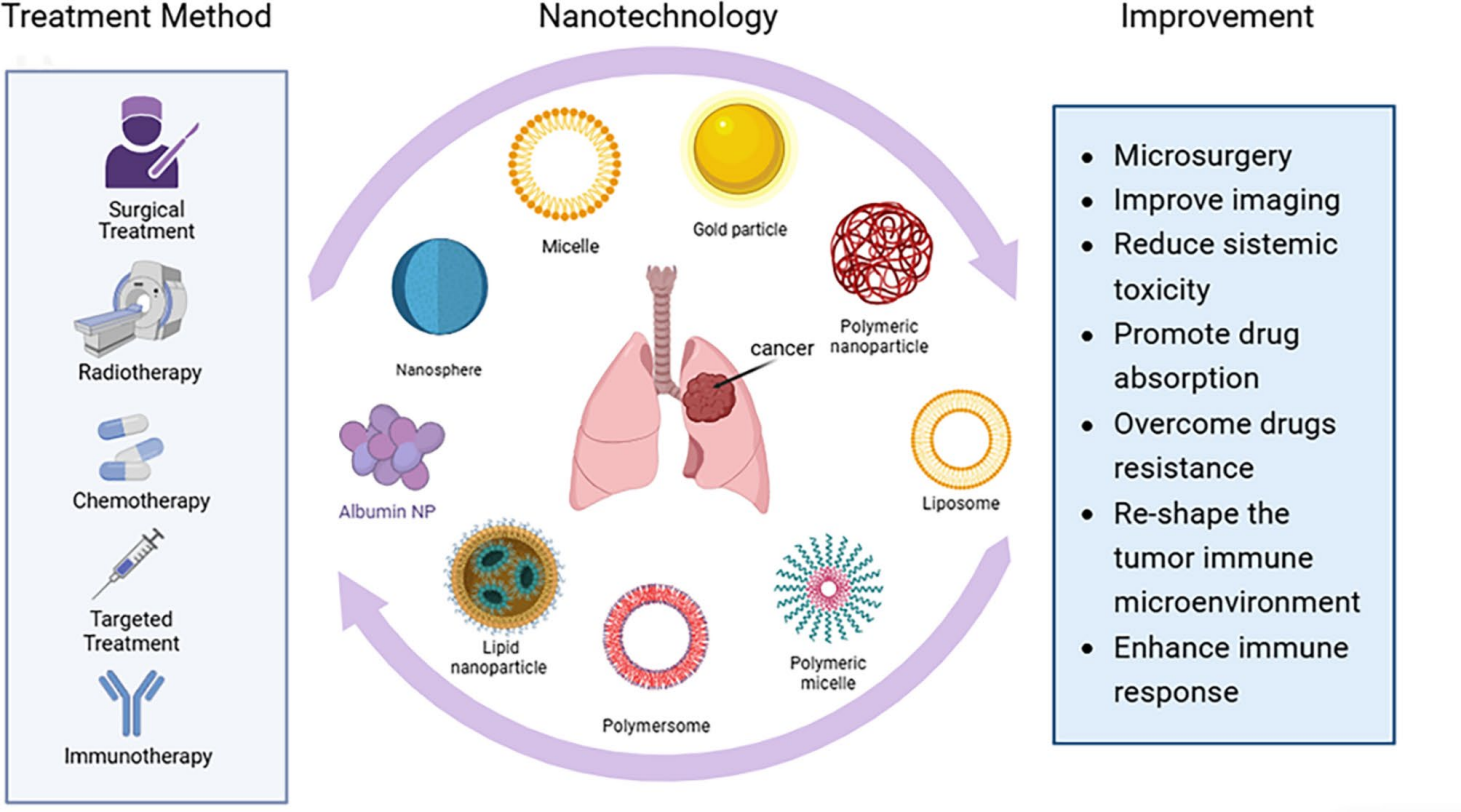
Research and application of nanotechnology in lung Cancer Treatment. Nanomaterials can surpass the limitations of current therapeutic methods and enhance treatment efficacy

**Fig. 5:**
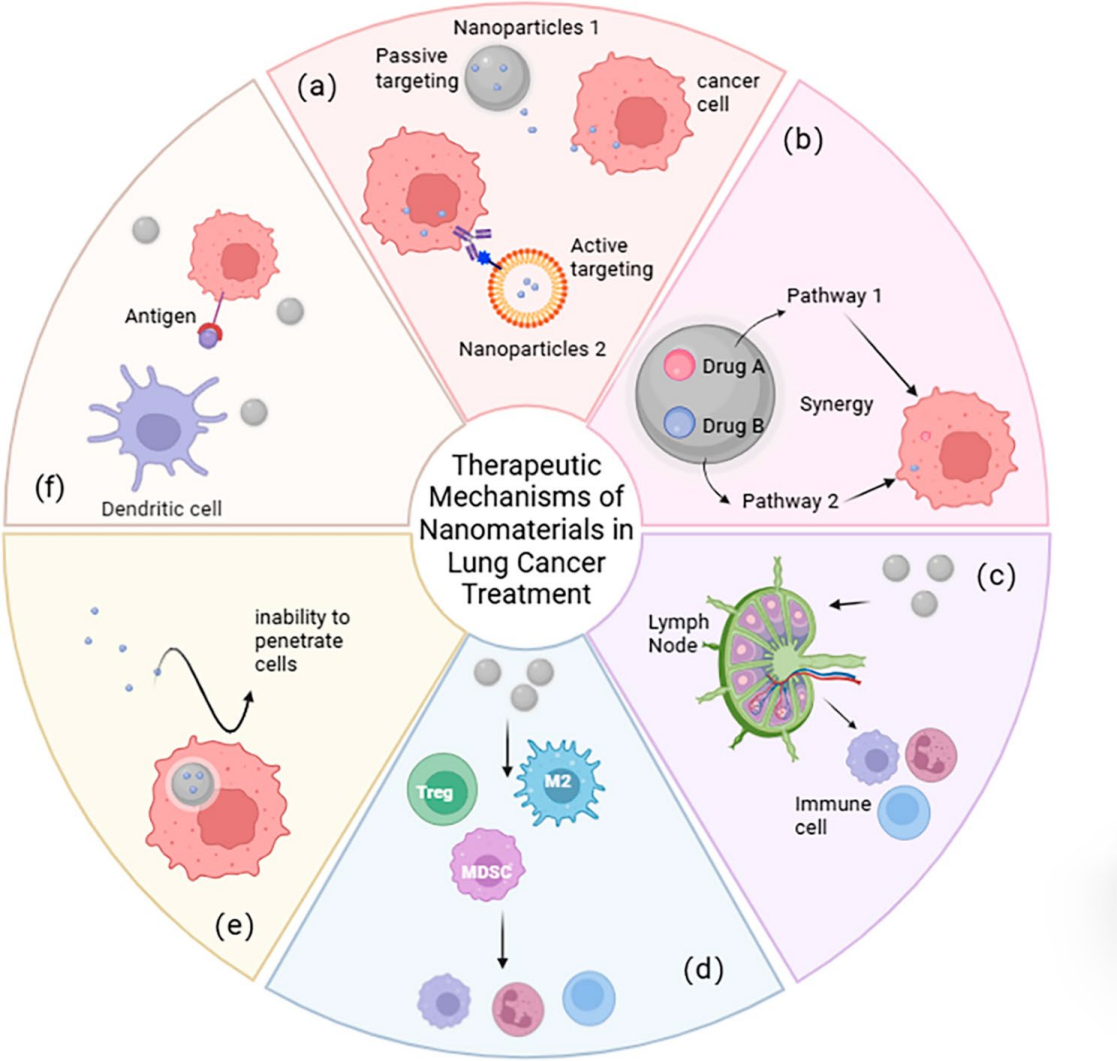
Therapeutic mechanisms of nanomaterials in lung Cancer. **a**) Reduce cytotoxicity. **b**) Overcome drug resistance. **c**) Activate immune responses. **d**) Create anti-tumor immune microenvironment. **e**) Promote drug enrichment. **f**) Enhance antigen presentation

The application of nanotechnology in the field of immunotherapy has not only improved drug delivery efficiency and immune activation levels but also provided new strategies and directions for overcoming the immunosuppressive tumor microenvironment and addressing the issue of resistance to immunotherapy (Figs. 4 and 5).

### Current status of nanotechnology in lung cancer clinical research

In the treatment of advanced non-small cell lung cancer (NSCLC), a pivotal multicenter Phase II clinical trial initiated in February 2018 has focused on treatment regimens following immune checkpoint inhibitor administration. The trial employed nab-paclitaxel, a nanoparticle albumin-bound paclitaxel, as monotherapy and conducted a comprehensive evaluation of its efficacy and safety. The results demonstrated an objective response rate (ORR) of 55.2%, with a disease control rate (DCR) as high as 86.2%. In terms of progression-free survival (PFS), the median was 5.6 months (95% CI, 4.4 to 6.7 months), and the 1-year and 2-year PFS rates were maintained at 34.5% and 13.3%, respectively. Additionally, the median overall survival (OS) was 11.9 months (95% CI, 0.8 to 23.0 months). In safety analysis, grade 3 or higher toxicities included leukopenia (27.6%), neutropenia (31.0%), peripheral sensory neuropathy (6.9%), elevated levels of alanine and aspartate aminotransferases (3.4%), and interstitial lung disease (3.4%). These results clearly indicate that after failure of PD-(L)1 inhibitor therapy, nab-paclitaxel not only improved ORR but also achieved a durable response rate of 13% in patients with advanced NSCLC, with toxicity reactions within an acceptable range [119]. Furthermore, on March 11, 2019, a Phase I study from Japan assessed the tolerability of carboplatin/nab-paclitaxel combined with thoracic radio-therapy in elderly patients with locally advanced NSCLC [120]. At the recommended dose, the treatment completion rate for 17 patients reached 100%, with the 80% confidence interval (CI) of 83.8–100%. The overall response rate was 76.5%, and the median PFS was 13.4 months, with a 95% CI of 4.2 to 21.4 months. Moreover, common grade 3 and 4 toxicities included leukopenia (23.5%), neutropenia (17.6%), anemia (5.9%), and infection (5.9%). Notably, one treatment-related death due to pneumonia was observed six months after the study ended. The comprehensive analysis indicates that the combination of carboplatin/nab-paclitaxel with thoracic radio-therapy shows not only good tolerability but also significant efficacy in elderly patients with locally advanced non-small cell lung cancer. On April 7, 2020, China initiated a phase II clinical trial aimed at evaluating the efficacy of camrelizumab in combination with nab-paclitaxel/platin-based chemotherapy as neoadjuvant therapy for resectable non-small cell lung cancer (NSCLC) [121]. Conducted across two hospitals, the study enrolled patients aged 18 to 70 with stage IIIA or IIIB (T3N2) NSCLC, randomly assigned to receive camrelizumab combined with chemotherapy or chemotherapy alone, followed by surgery. The results demonstrated that the pathological complete response (pCR) rate in the camrelizumab plus chemotherapy group was 32.6% (14 out of 43; 95% CI, 19.1-48.5%), significantly higher than the 8.9% (4 out of 45; 95% CI, 2.5-21.2%) in the chemotherapy-only group, with an odds ratio of 4.95 (95% CI, 1.35–22.37; *P* = .008). The major pathological response (MPR) rate in the camrelizumab plus chemotherapy group was 65.1% (95% CI, 49.1-79.0%), compared to 15.6% (95% CI, 6.5-29.5%) in the chemotherapy-only group. The objective response rate (ORR) by imaging was 72.1% (95% CI, 56.3-84.7%) in the camrelizumab plus chemotherapy group and 53.3% (95% CI, 37.9-68.3%) in the chemotherapy-only group. With a median follow-up time of 14.1 months (IQR, 9.2–20.9 months), neither group reached the median event-free survival (EFS) and disease-free survival (DFS). The most common grade 3 or higher adverse events in the camrelizumab plus chemotherapy group were decreased white blood cell count and neutrophil count, with no treatment-related deaths reported. The study confirms that the combination of camrelizumab with nab-paclitaxel/platin-based chemotherapy has a significant advantage in improving the pathological response rate in patients with resectable NSCLC, with a favorable safety profile (Table 2).

**Table 2:** Clinical trials related to nanomedicines in lung cancer (completed)

Clinical trial identifier	Phase	Start date	Status	Cancer type (population, *N*)	Interventions and combination	Primary outcome measures	Secondary outcome measures
UMIN000030994	II	February, 2018	Complete	NSCLC (*N* ≈ 30)	PD-(L)1 Inhibitors + Nab-Paclitaxel	ORR (55.2%)	DCR (86.2%), Median PFS (5.6 months), Median OS (11.9 months), Safety
UMIN000014764	I	March 11, 2019	Complete	NSCLC (*N* ≈ 19)	Nab-Paclitaxel + Carboplatin + Thoracic Radiotherapy (TRT)	FDLT, TCR(100%)	Safety, RR (76.5%), Median PFS (13.4 months), 2-year Survival Rate, OS, Site of First Disease Progression
NCT04338620	II	April 07, 2020	Complete	NSCLC (*N* ≈ 94)	Experimental Group: camrelizumab + Nab-Paclitaxel + platinum. Control group: Nab-Paclitaxel + platinum.	pCR	MPR, ORR, EFS, AEs

a)**NSCLC**,Non-Small Cell Lung Cancer; b)**PD-(L)1**,Programmed Death-1 (PD-1) and its ligands; c)**Nab-Paclitaxel**,Albumin-bound Paclitaxel; d)**ORR**,Objective Response Rate; e)**DCR**,Disease Control Rate; f)**Median PFS**,Median Progression-Free Survival; g)**Median OS**,Median Overall Survival; h)**FDLT**,First Disease Location at Time; i)**TCR**,Total Complete Response; j)**RR**,Response Rate; k)**OS**,Overall Surviv; l)**pCR**,Pathological complete response rate; **m)AEs**,Adverse Events; n)**EFS**,Event-Free Survival

Clinical studies combining nanotechnology with traditional therapeutic approaches for lung cancer have preliminarily demonstrated their efficacy and safety. A series of clinical studies are currently underway to assess the safety and efficacy of nanotechnology in combination with other methods for treating lung cancer (Table 3).

**Table 3:** Clinical trials related to nanomedicines in lung cancer (on-going)

Clinical trial identifier	Phase	Start date	Status	Cancer type (population, *N*)	Interventions and combination	Primary outcome measures	Secondary outcome measures
ChiCTR2300068627	II	November 1, 2022	Unknown	SCLC	Nab-Paclitaxel +Anlotinib	PFS	OS, DCR, ORR, Safety
ChiCTR2300072520	IV	June 30, 2023	Not Recruiting	SCLC	Adbelimab + Apatinib Mesylate + Nab-Paclitaxel	1-year Overall Survival Rate	ORR, PFS, OS, 6-month survival rate, duration of therapy
ChiCTR2300077241	II	November 2, 2023	Not Recruiting	Lung cancer	Cadonilimab (AK104) + Nab-Paclitaxel + Platinum-based chemotherapy	6-month Progression-Free Survival Rate	PFS, OS, 6-month survival rate, 1-year Overall Survival Rate, ORR, QOL, AEs
ChiCTR2300077236	II	November 2, 2023	Recruiting	Lung cancer	Envafolimab + Nab-Paclitaxel + Platinum-based chemotherapy	6-month Progression-Free Survival Rate	PFS, OS, ORR, quality of life, AEs
ChiCTR2300078968	IV	November 30, 2023	Recruiting	SCLC	Group A: Ateolimab + Apatinib + Nab-Paclitaxel Group B: Ateolimab + Apatinib Group C: Apatinib + Nab-Paclitaxel	ORR	OS, DCR, DOR, Progression-Free Survival Adverse Events, Serious AEs, Tumor Markers, QOL
ChiCTR2400082754	II	April 15, 2024	Not Recruiting	NSCLC	Atezolizumab + Nab-Paclitaxel + Platinum-based chemotherapy	Pathologic Complete Response Rate	Pathologic Complete Response Rate, R0 Resection Rate, Event-Free Survival, OS, ORR, Safety
ChiCTR2400083693	II	March 21, 2024	Recruiting	SCLC	Group A: Irinotecan Liposome (II) +Famitinib Group B: Irinotecan Liposome (II) + platinum-based chemotherapy Group C: Irinotecan Liposome (II)	PFS	6-month Progression-Free Survival Rate, OS, ORR, DCR, DOR, Safety
CRB7180005	II	April 27, 2022	Not Recruiting	TTF-1 negative treatment-naive advanced non-squamous non-small cell lung cancer	Atezolizumab + Carboplatin + Nab-Paclitaxel	PFS	OS, Response proportion, DOR, TTF, Dose intensity/Relative dose intensity, Safety
CRB4180010	II	June 10, 2021	Recruiting	NSCLC	Atezolizumab + Carboplatin + Nab-Paclitaxel	ORR	Safety, Tolerability, OS, DOR, PFS, 6-month Progression-Free Survival Rate, PS Improvement Rate
jRCTs071200102	II	February 25, 2021	Not Recruiting	NSCLC, advanced, Renal dysfunction NSCLC	Atezolizumab + Carboplatin + Nab-Paclitaxel	RR	PFS, OS, DOR, TTF, Dose intensity/Relative dose intensity, Safety
jRCTs041180110	III	March 19, 2019	Recruiting	Elderly patients with advanced squamous non-small-cell lung cancer	Group A: Docetaxel Group B: Carboplatin + Nab-Paclitaxel	OS	PFS, RR, Toxicity, QOL
jRCTs042180077	I	November 11, 2019	Complete Recruiting	NSCLC, unresectable NSCLC	CBDCA + Nab-Paclitaxel	Frequency of Dose-Limiting Toxicity, Treatment Completion Rate	Safety, RR, PFS, 2-year survival rate, OS site of first disease progression
jRCTs071180037	III	November 11, 2019	Complete Recruiting	NSCLC	Group A: Nab-Paclitaxel Group B: DTX	OS	PFS, RR, Toxicity, QOL
jRCTs031180214	II	March 8, 2019	Complete Recruiting	advanced non-small cell lung cancer	Group A: Nab-Paclitaxel (100 mg/m^2^) Group B: Nab-Paclitaxel (70 mg/m^2^)	PFS	OS, ORR, AEs
ChiCTR2400085724	I	June 30, 2024	Prospective registration	NSCLC	Carboplatin + Nab-Paclitaxel	PFS	OS, ORR, DOR, DCR, iPFS, iORR
NCT 04826913		February 25, 2021	Unknown	NSCLC	Device: Organoids	Testing of anti-cancer drugs [ Time Frame: 3 years]	
NCT03967652		March 19, 2019	Unknown	Cancer	Diagnostic Test: Sensor based on Nanomaterials	Establishment of a Predictive Diagnostic Database [Time Range: From July 1, 2019, to December 31, 2021]	Exhaled breath-related characteristics associated with differentially expressed genes [Approximate time: from January 1, 2022 to December 31, 2022]
NCT06421298	II	May 17, 2024	Recruiting (*N* ≈ 30)	Lung Cancer	Nab-Paclitaxel + Sintilimab + Gemcitabine + Docetaxel	PFS	OS, DCR, DOR, ORR, AEs
NCT06357598	III	January 18, 2024	Recruiting (*N* ≈ 30)	NSCLC	Surgery + Carboplatin or Cisplatin + Pemetrexed (Non-squamous NSCLC) or Nab-Paclitaxel(Squamous NSCLC) + Tislelizumab	R0 Resection Rate	ORR, Resectability Rate, MPR Rate, Rate of grade 3 and higher grade treatment-related adverse events, PFS
NCT06299371	II	April 15, 2024	Not Recruiting	NSCLC	Adebrelimab + Cisplatin or Carboplatin + Nab-Paclitaxel	pCR	MPR Rate, R0 rate, EFS, OS, ORR, AEs
CTR20231113	III		Not Recruiting	NSCLC, Metastatic non small cell lung cancer	Experimental: Carboplatin + Pemetrexed Disodium + Cisplatin + Paclitaxel + MK-3475 A + Nab-Paclitaxel Comparator: Pembrolizumab + Carboplatin + Pemetrexed Disodium + Cisplatin + Paclitaxel + Nab-Paclitaxel		ORR, PFS, OS, DOR, AEs
NCT05766800	II	March 14, 2023	Recruiting (*N* ≈ 100)	Locally Advanced non small cell lung cancer	Experimental: Serplulimab + Liposomal paclitaxel + Pemetrexed + Surgery + Carboplatin + Nab-Paclitaxel Comparator: Serplulimab + Liposomal paclitaxel + Pemetrexed + Radiotherapy + Carboplatin + Nab-Paclitaxel	EFS	ORR, MPR, PFS, DFS, OS, DOR, DCR, R0 Rate, SAE, QOL
NCT05407155		June 01, 2022	Not Recruiting (*N* ≈ 56)	Metastatic NSCLC, Non- squamous non small cell Lung	bevacizumab + Nab-Paclitaxel + platinum	ORR	PFS, OS
NCT04865250	II	January 07, 2021	Recruiting (*N* ≈ 20)	NSCLC Stage II, NSCLC, Stage IIIA, NSCLC Stage IIIB	Atezolizumab; Carboplatin; Nab-Paclitaxel	MPR	EFS, OS,
NCT04213937	II	January 31, 2020	Unknown (*N* ≈ 386)	Extensive Stage small cell lung cancer	Experimental: Nab-paclitaxel Comparator: Topotecan	OS	ORR, PFS, AEs
NCT04056949	II	August 05, 2019	Unknown (*N* ≈ 30)	Extensive Stage small cell lung cancer	IBI308 + Nab-Paclitaxel	PFS	OS, DCR, DOR, ORR
NCT04725448	II	April 06, 2021	Unknown (*N* ≈ 27)	NSCLC	Toripalimab + Bevacizumab + Nab-Paclitaxel + Carboplatin	PFS	OS, ORR, DCR, QOL, AEs
NCT04541251	II	August 01, 2020	Unknown (*N* ≈ 27)	Lung cancer, NSCLC	Camrelizumab + Nab-Paclitaxel + Carboplatin	MPR	pCR, ORR, DFS, R0, OS, AEs, SAEs, QOL
NCT04015778	II	August 08, 2019	Unknown (*N* ≈ 48)	NSCLC	Experimental 1: Nivolumab Experimental 2: Nivolumab + carboplatin + Nab-Paclitaxel	MPR	Proportion of resection without delay, AEs
NCT04789486	I/II	May 27, 2021	Recruiting	NSCLC	Phase I Experimental: AGUIX(Injected gadolinium-based nanoparticles) + SMART Phase II Experimental: AGUIX + SMART Experimental: SMART	I: MTD(Time Frame: 3 months) II: MTD(Time Frame: 12 months)	Progression-free survival (PFS) at Maximum tolerated dose (MTD), Overall Response Rate (ORR) at Maximum tolerated dose (MTD), Serious Adverse Events at 90 Days etc.
NCT06028633	II	October, 2023	Not Recruiting (*N* ≈ 28)	Advanced Non-squamous Non-small-cell Lung Cancer, Non-Squamous Non-Small Cell Lung Cancer, Metastatic Non-squamous Non Small Cell Lung Cancer	Pembrolizumab + Lenvatinib + Nab-Paclitaxel	ORR	OS, PFS, DOR, AEs

a)**TTF-1**,Thyroid Transcription Factor-1; b)**PFS**,Progression-Free Survival; c)**OS**,Overall Survival; d)**DCR**,Disease Control Rate; e)**ORR**,Objective Response Rate; f)**DOR**,Duration of Response; g)**TTF**,Time to Treatment Failure; h)**QOL**,Quality of Life; i)**AEs**,Adverse Events; j)**MTD**,Maximum Tolerated Dose; k)**RR**,Response Rate; l)**MTD**,Maximum Tolerated Dose; m)**pCR**,Pathological complete response rate; n)**HRQol**, Health related quality of life; o)**DLTs**, dose-limited toxicities, p)**CRR**,Clinical response rate; q)**R0**,complete resection rate; r)**SAEs**,Serious adverse events; **s)EFS**,Event-Free Survival; t)**Nab-Paclitaxel**,Albumin-bound Paclitax

## Summary and prospects

The research and application of nanomaterials in the field of cancer diagnosis and therapy is becoming increasingly widespread and diverse. This review focuses on several types of nanomaterials that have been extensively studied in lung cancer research, including lipid-based, protein-based, polymer-based, and metal-based nanomaterials. Other nanomaterials not mentioned in this article, such as hybrid nanomaterials and biomimetic nanomaterials, also play a very important role in the field of lung cancer diagnosis and therapy. The integration of nanotechnology has injected new vitality into the research of early lung cancer screening and diagnosis. In terms of diagnosis, nanoparticles as contrast agents, nano-probes, and biosensors can significantly enhance the sensitivity and specificity of lung cancer detection. The non-invasive cancer marker capture technology of biosensors effectively complements the deficiencies of traditional invasive diagnostic methods. Notably, the dual role of nanoparticles in lung cancer diagnosis—tracing and therapy—provides the possibility for the integration of diagnosis and therapy. In terms of therapy, nanotechnology has also shown great potential in enhancing the efficacy of radio-therapy and chemotherapy. By using nanocarriers for oxygen loading, enhancing the radiosensitivity of tumor tissues, and accurately delivering radioactive isotopes, it reduces the toxicity to normal tissues. In the enhancement of chemotherapy drug efficacy, nanotechnology is committed to reducing systemic cytotoxicity, sensitizing chemotherapy drugs, and overcoming drug resistance. Research on nanotechnology in targeted lung cancer therapy aims to achieve co-delivery or multi-drug delivery of targeted drugs through nanocarriers to address the problem of drug resistance in targeted therapy. Research on nanotechnology in immunotherapy aims to enhance immune activation and improve the immunosuppressive microenvironment, providing effective strategies for treatment through the combined use of various treatment methods or co-delivery of drugs targeting different immune targets. In clinical trials, the combination of nanotechnology with traditional therapy has achieved significant therapeutic effects, not only improving the objective response rate (ORR) and the rate of durable remission but also demonstrating good tolerance. Surgical treatment, radio-therapy, chemotherapy, targeted therapy, and immunotherapy, as the main treatment methods for lung cancer, have been widely applied in clinical practice and are becoming increasingly mature. Although methods such as photodynamic therapy (PDT), sonodynamic therapy (SDT), and photothermal therapy (PTT) have relatively limited clinical applications, their advantages and research value should not be overlooked. The integration of nanotechnology is expected to deepen the research and application of these treatment methods in the future.

However, the clinical translation of nanomedicine is not without challenges. Issues such as biocompatibility and safety concerns, the complexity of manufacturing and quality control, optimization of drug delivery efficiency, understanding of nano-bio interface interactions, assessment of long-term side effects and cumulative toxicity, cost issues, the complexity of clinical trial design, and drug resistance are all challenges that must be faced in the clinical translation of nanomedicine. Despite these difficulties, with technological advancements and in-depth research, these issues are expected to be gradually resolved. Through innovation in materials science, optimization of production processes and quality control, in-depth study of nano-bio interface interactions, assessment through long-term clinical studies, cost reduction through scaled production and technological innovation, and well-designed clinical trials, the prospects for clinical application of nanomedicine will become increasingly promising.

Circulating tumor DNA (ctDNA) detection is one of the most clinically significant methods for early cancer diagnosis within liquid biopsy [122, 123]. On June 12, 2024, JAMA Oncology published another significant outcome of the ctDNA-MRD monitoring applied to the comprehensive management of lung cancer treatment by Professor Wu Yilong’s team [124]. This study is the first to propose the concept of ctDNA-guided adaptive de-escalation therapy in the field of lung cancer, showing a promising prospect. Recently, a new strategy published in Science has provided a new direction for ctDNA detection. The free ctDNA in the bloodstream is mainly affected by the clearance of liver-resident macrophages and the degradation of circulating nucleases, resulting in only trace amounts of ctDNA present in the blood, which limits the sensitivity of liquid biopsy. Researchers have developed two types of primers: one is a nanoparticle primer that inhibits the phagocytosis of ctDNA by liver-resident macrophages, and the other is a DNA-binding monoclonal antibody (mAb) that protects ctDNA from degradation by circulating nucleases. These studies have shown potential in improving the recovery rate of ctDNA and the sensitivity of detecting small tumors in a colorectal cancer model [125, 126]. Of course, there are still many issues that are not clear about the strategy of detecting ctDNA based on these two primers, such as the cytotoxicity caused by these two primers, which is the key to whether this strategy can be clinically translated; there is also the issue of the scope of application of this strategy, whether it can be used in lung cancer or other types of cancer?

The rapid rise of nanorobot technology has brought revolutionary changes to the field of nanotechnology [127]. As drug delivery carriers and nanoscale surgeons, nanorobots can not only deliver drugs but also enter the human bloodstream, cells, or even more subtle and hidden parts of the body to perform microsurgeries [128]. The synthesis of nanorobots for non-invasive vascular interventional surgery to deliver thrombin to form thrombi, cutting off the connection between tumors and blood vessels, assisting in surgical operations, and in the future, nanorobots may be able to achieve integrated functions of disease monitoring, diagnosis, treatment, and immune memory in the human body, and automatically degrade and expel or safely hibernate after completing the task [129].

In summary, various types of nanomaterials and nanotechnologies are gradually being valued and applied in different pathological types of lung cancer according to their different advantages and disadvantages. The research on nanomaterials has preliminarily shown its great application potential in the early screening, diagnosis, treatment, and post-treatment MRD monitoring of lung cancer. Although there are still many challenges in the clinical translation of nanotechnology in the field of lung cancer diagnosis and treatment, with in-depth research and technological maturity, we have reason to believe that nanotechnology will bring a brighter future for lung cancer treatment.

## Data Availability

No datasets were generated or analysed during the current study.

## Abbreviations

ADCs: Antibody-Drug Conjugates
ALB-NPs: Albumin Nanoparticles
Ad: Adenoviruses
AEs: Adverse Events
BSA: Bovine Serum Albumin
CMNP: Cell Membrane-Camelid Nanoparticle
CP: Capsular Polysaccharide
CRISPR/Cas9: Clustered Regularly Interspaced Short Palindromic Repeats/CRISPR-associated protein 9
CRTX: Cetuximab
CT: Computed Tomography
CRR: Clinical Response Rate
DCR: Disease Control Rate
DLTs: Dose-Limiting Toxicities
DM@NPs: Doxorubicin-Membrane-Coated Nanoparticles
DOR: Duration of Response
ECL-RET: Electrochemiluminescence Resonance Energy Transfer
EFS: Event-Free Survival
EGFR: Epidermal Growth Factor Receptor
EGFR-TKI: Epidermal Growth Factor Receptor Tyrosine Kinase Inhibitor
EPR: Enhanced Permeability and Retention
NES: Neuron-Specific Enolase
ExtraCRAd: Extra Cellular Adenovirus-Derived Nanoparticles
FDA: Food and Drug Administration
FeMOFs: Iron-based Metal-Organic Frameworks
GaTa-CP NP: Gallium-Tannic Acid-Capsular Polysaccharide Nanoparticle
GSDMD: Gasdermin D
HRQoL: Health-Related Quality of Life
HRV2: Human Rhinovirus Type II
HSA: Human Serum Albumin
ICD: Immunogenic Cell Death
IRES: Internal Ribosome Entry Site
LDCT: Low-dose Computed Tomography
LDI-TOF MS: Laser Desorption Ionization Time-of-Flight Mass Spectrometry
LNPs: Lipid Nanoparticles
MPR: Major Pathologic Response
MRI: Magnetic Resonance Imaging
MTD: Maximum Tolerated Dose
NSCLC: Non-Small Cell Lung Cancer
NSE: Neuron-Specific Enolase
OLA: Oleic Acid
ORR: Objective Response Rate
OS: Overall Survival
PBAE: Poly(beta-amino ester)
PD-L1: Programmed Death-Ligand 1
PEG: Polyethylene Glycol
PFCE NPs: Perfluoro-15-crown-5-ether Nanoparticles
PNPs: Polymeric Nanoparticles
PPE: Polyethylene Glycol-modified PAMAM
PTT: Photothermal Therapy
QOL: Quality of Life
R0: Complete Resection Rate
RR: Response Rate
SAEs: Serious Adverse Events
SCLC: Small Cell Lung Cancer
siRNA: Small Interfering RNA
TRT: Thoracic Radio-therapy
UCILA: Upconversion Nanoparticles in Lipidic Aptamer Nanostructures
UCNPs: Upconversion Nanoparticles
USRP: Ultra-Small Rigid Platforms
UTE-MRI: Ultra-Short Echo Time Magnetic Resonance Imaging
VLPs: Virus-Like Particles
